# Exploring non-assembly 3D printing for novel compliant surgical devices

**DOI:** 10.1371/journal.pone.0232952

**Published:** 2020-05-14

**Authors:** Costanza Culmone, Paul W. J. Henselmans, Remi I. B. van Starkenburg, Paul Breedveld

**Affiliations:** 1 Department BioMechanical Engineering, Bio-Inspired Technology Group (BITE), Faculty of Mechanical, Maritime, and Materials Engineering, Delft University of Technology, Delft, The Netherlands; 2 Department of Electronic and Mechanical Support Division, Delft University of Technology, Delft, The Netherlands; Boston University, UNITED STATES

## Abstract

In minimally invasive surgery, maneuverability is usually limited and a large number of degrees of freedom (DOF) is highly demanded. However, increasing the DOF usually means increasing the complexity of the surgical instrument leading to long fabrication and assembly times. In this work, we propose the first fully 3D printed handheld, multi-steerable device. The proposed device is mechanically actuated, and possesses five serially controlled segments. We designed a new compliant segment providing high torsion and axial stiffness as well as a low bending stiffness by merging the functions of four helicoids and a continuum backbone. Compliant segments were combined to form the compliant shaft of the new device. In order to control this compliant shaft, a control handle was designed that mimics the shaft structure. A prototype called the HelicoFlex was built using only three 3D printed parts. HelicoFlex, with its 10 degrees of freedom, showed a fluid motion in performing single and multi-curved paths. The multi-steerable instrument was 3D printed without any support material in the compliant shaft itself. This work contributes to enlarge the body of knowledge regarding how additive manufacturing could be used in the production of multi-steerable surgical instruments for personalized medicine.

## Introduction

Over the last decades, one of the most significant innovations in surgery is the transition from open surgery to minimally invasive surgery (MIS). In open surgery, the area of interest is directly exposed, and depending on the specific procedure, the incision can be relatively large [1][2]. However, increasing the size of the incision means increasing the risk of infection as well as the recovery time for the patient [3][4]. Conversely, MIS strives to reduce the incision size by using smaller instruments and indirect visualization using endoscopes. However, using conventional tools that are straight, long, and rigid while decreasing the size of the incision, has a high impact on the maneuverability of the instruments and the reachability of the area of interest. The problem is evident in *Natural Orifice Transluminal Endoscopic Surgery* (NOTES) which uses natural orifices such as mouth, nose, or anus as the entry port of the body [5][6]. For instance, *Endoscopic Endonasal Surgery* (EES) is a NOTES procedure in which the nostrils are the entry port to reach and remove tumors at the base of the skull. A common procedure is the removal of adenomas in the pituitary gland. The narrow corridor through the nostrils limits the maneuverability of the used rigid instruments.

**Fig 1:**
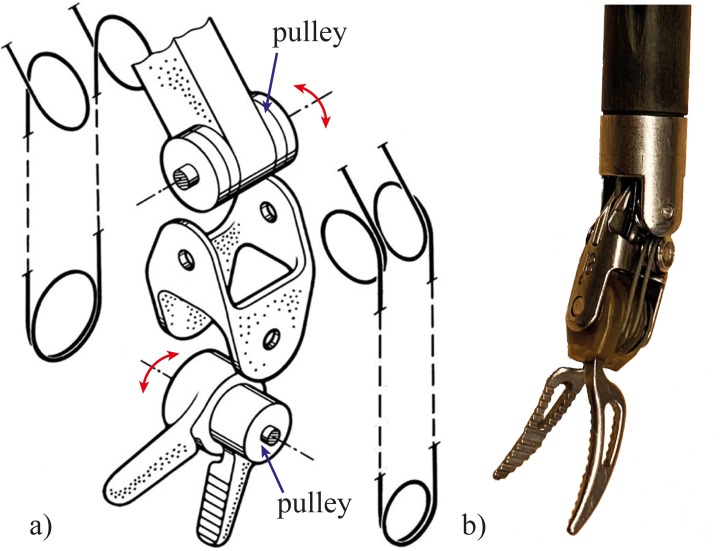
Endo Wrist Grasper. a) Schematic exploded view of the Endo Wrist in which the different driving cables, pulleys, and rivets are shown, adapted from Breedveld et al. [9], based on the EndoWrist patent [10], and b) a Ø 8 mm Endo Wrist Grasper. In the EndoWrist, the two pulleys are positioned perpendicular to each other provide two DOF (red arrows).

Research groups have analyzed the problem of instrument maneuverability, and many devices have been proposed [7]. The use of the well-known da Vinci^®^ robotic system (Intuitive Surgical Inc., Sunnyvale, Ca, USA) with integrated EndoWrist technology: a two degrees of freedom (DOF) mechanism for steering into the distal end of the surgical instruments, increases surgeon dexterity in a laparoscopy scenario allowing procedures in which high maneuverability is required [8] (Fig 1).

However, steerability is still limited due to rigid instrument shafts which do not permit navigation through curves with multiple radii [10]. Moreover, miniaturized pulleys, which are at the base of the EndoWrist design, guide the cables, provoking fatigue and limiting the lifespan to a maximum of ten sterilization cycles, increasing the overall costs [11].

In order to overcome the rigidity of the shaft, research has been focused on the integration of an additional flexible component at the end of the rigid shaft, allowing maneuvering over more complex curvatures and expanding the motion beyond the 2 DOF EndoWrist. The flexibility of the additional component can be obtained using continuum structures. Multi-steerable instruments based on continuum structures can be compared with “invertebrates” due to their ability to form continuous curves [12][13]. Examples of continuum structures are concentric telescopic tube robots [14] composed of concentric precurved tubes placed into one another. The control of the rotation and the translation of the tubes relative to each other allows the formation of curved shapes [15][16]. Another approach is using a single- or a multi-backbone structure. In a single-backbone structure, a single element supports the entire flexible segment. This element can be made of shape memory alloys [17], springs [18], flexible tubes [19], or variable stiffness mechanisms [20][21]. Multi-backbone structures are based on multiple elements, for example parallel rods, that equally contribute to the motion of the flexible tip [22].

Regardless of the number of backbones used, multi-steerable instruments require a method of actuation. Types of actuation are based on hydraulic and pneumatic principles, as well as shape memory alloys [7]. However, tendon-driven actuation remains most commonly used in medical applications due to the possibility of minimizing the size of the tip while at the same time controlling a large number of DOF [23]. In an attempt to control the complex motion of such a multi DOF shaft, each DOF can be individually controlled and actuated using independent electric motors. Due to their relatively large size, these motors should then be placed outside the patient at the control side of the instrument. However, the use of a large number of motors generally results in high production costs, difficulties in sterilization, and unsuitability for disposable use [24]. Moreover, a system with a large number of motors requires a large footprint, reducing the workspace for the surgeon near the patient.

In an attempt to solve these problems, manually actuated instruments such as the HelixFlex proposed by Gerboni *et al*. or the multi-backbone elbow device presented by Riojas *et al*., have been developed, presenting a completely different approach by being multi-steerable and at the same time handheld [25][26]. These instruments are fully mechanically actuated with no need for electric motors. Fan *et al*. [27] give a comprehensive overview of handheld (multi-)steerable instruments and Anderson *et al*. [28] of their control methods. Although the proposed handheld devices meet the requirements in terms of flexibility, miniaturization, and maneuverability, they are still very complex, containing numerous complex-shaped parts, impairing the assembly process, and making the device unsuited for sterilization or low-cost disposable use.

Additive manufacturing (AM), also referred to as three-dimensional (3D) printing, might provide a solution. AM enables a computer-aided design (CAD) to be directly converted into a 3D object with a layer by layer printing process. AM allows the production of structures with complex geometries that cannot be produced with conventional fabrication techniques. Moreover, this increase in geometrical complexity allows for the integration of more functionality into a single part, consequently reducing the need for assembling multiple parts. Many research groups are exploiting this technology in the field of medical instruments [29]. An example is given by the manipulator presented by Mintenbeck *et al*. in which AM was used in the fabrication of the steerable segments [30]. Morimoto *et al*. applied AM into concentric tube robots investigating different materials and 3D printing technology [31], whereas at the Technical University of Munich researchers developed a 3D printed overtube to enhance the properties of conventional flexible endoscopes [32][33].

Although problems such as steerability and miniaturization have been addressed, design complexity is still high and the number of components is still large, hindering the reduction of assembly time. Therefore, this study explores the use of AM for the development of a manually actuated tendon-driven multi-steerable surgical device, intending to simplify its fabrication and assembly process to make it suitable for disposable use. A new device called HelicoFlex was developed as a first explorative case to combine easy manufacture with very high steering performance. HelicoFlex is the first handheld device that is printed in only three parts with five steerable segments, which enables the control of 10 DOF. In the first part of the paper, we will explore new geometries to find a design paradigm to combine the characteristics of a compliant shaft and minimize the number of parts while using AM. In the second part of the paper, we present the entire design and study its behavior in performing complex curves.

## Conceptual design of the compliant shaft

### Design requirements

The compliant shaft of our instrument should allow high steerability in terms of *multiple DOF* to follow tortuous paths and complex curves with different radii. The device should, therefore, include multiple segments that are serially connected and each bendable in 2 DOF. Each segment should have a *high axial and torsional stiffness*, whereas a relatively *low bending stiffness* is preferred. Axial stiffness is required for reliable control of the compliant shaft whereas torsional stiffness is required to endure axial torques that arise from external loads. On the contrary, low bending stiffness is preferred as it improves the bendability of the entire shaft and limits the required tensile forces on the steering cables, reducing the forces required for actuation and resulting in lower friction forces in the system. For application in MIS, the *diameter* of the compliant shaft should not exceed 10 mm [34], integrating at least one *lumen* to allow for the insertion of additional instruments (e.g., biopsy forceps) or tools to visualize and operate on the area of interest. *Guidance and fixation of cables* are two of the most challenging functionalities within a tendon-driven device, and therefore often have a significant influence on the shape, fabrication, and assembly process of the device, especially in multi-steerable instruments that incorporate many cables. An effective and scalable method for integrating the functionalities of cable guidance and fixation in preferably one single component was therefore a key research topic in the design of our instrument. Finally, the new compliant shaft should preferably be 3D printed *without support* material, which is sacrificial material needed to print specific overhangs, in order to reduce the post-processing time after the printing process.

### Design choices

Conventional steerable instruments are generally based on a chain of connected rigid elements. In the specific case of EndoWrist, steering in two directions is provided by a series of miniature pulleys placed perpendicular to each other and individually controlled by driving cables looped around the pulleys (Fig 1). As the diameter of the pulleys is too small as compared to the thickness of the driving cables, the cables suffer from fatigue, reducing the lifespan of the EndoWrist to only ten procedures [35]. In our design, we aimed at avoiding pulleys by using a cable guidance system that does not generate fatigue. Moreover, we aimed at greatly expanding EndoWrist’s motion to 10 DOF while merging all its rigid-linked frame properties into one 3D printed compliant component without using support, combining high axial and torsional stiffness with low bending stiffness. We strived to have at least one lumen and simple means to guide and fix 20 actuation cables, to facilitate fast and easy assembly.

#### Compliant segment design

**Fig 2:**
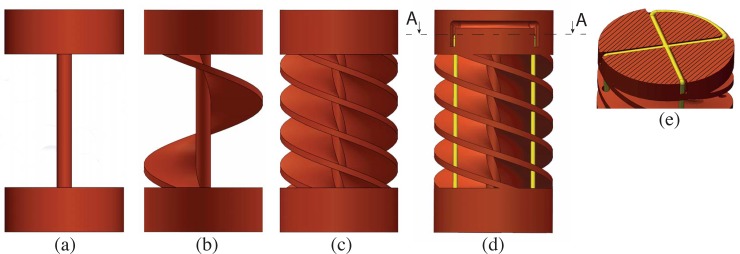
Schematic drawing of a 3D-printed compliant shaft segment with actuation cables in yellow. (a) a solid backbone gives a high axial stiffness; (b) a helicoid increases torsion stiffness; (c) increasing the number of helicoids makes the system more homogeneous; (d) holes are added to guide four actuation cables through the helicoids; and (e) the segment is completed with an additional structure to loop and fasten the actuation cables.

A compliant structure that combines high axial stiffness with low bending stiffness can be created by using a thin beam serving as a *continuous backbone* at the center of a steering segment (Fig 2A).

However, a thin beam is not torsion stiff. Increasing the diameter of the beam would provide higher torsion stiffness, yet would also increase its bending stiffness. Thus, an additional element has to be added to ensure torsion stiffness. A *helicoid* is a compliant element able to provide high torsion stiffness and low bending stiffness. In our design, we decided to combine these two elements: a continuous thin central backbone around which a helicoid runs (Fig 2B). Four helicoids were evenly placed around the centerline to provide a more homogeneous torsion stiffness as compared to only one helicoid (Fig 2C). The pitch of each helicoid was kept equal to the length of the backbone, meaning that each helicoid makes one full turn within the length of the backbone.

**Fig 3:**
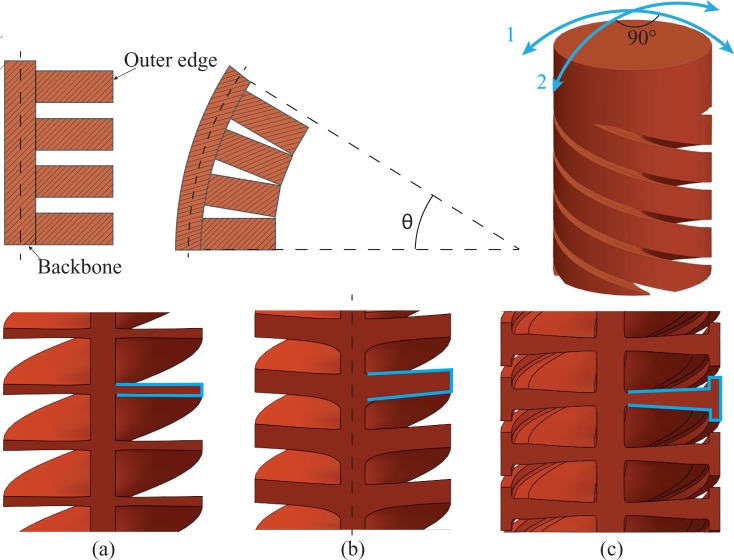
Helicoids shape design. Top: Sketch of a compliant segment in a straight and bent position with light blue arrows showing the two DOF per segment; each segment being able to bend in two perpendicular directions. Bottom: different shapes of the helicoids cross-section: (a) thin rectangular shape, (b) thick rectangular shape, (c) T-shape.

Finally, we defined the exact shape of the four helicoids. As shown in the cross-section in Fig 3A, we started with a thin rectangular shape for the helicoids. However, at the inside of these helicoids, cracks can be provoked by excessive bending. By increasing the thickness of the helicoids, we can ensure that the helicoids would touch each other at their outer edge, thus creating a stop for the bending (Fig 3B). However, increasing the thickness of the helicoid at the outer edge while keeping the rectangular shape would increase the bending stiffness as, in this case, more material is added to the backbone. Therefore, we decided to change the rectangular shape into a T-shape (Fig 3C), keeping a low bending stiffness while at the same time limiting the maximum bending angle.

#### Cable fixation method

As previously discussed, cable fixation is an important aspect that can affect the robustness of the device, causing malfunctioning or breakages. In our compliant design, we decided on an alternative *cable fixation method* avoiding soldering or gluing in the shaft. Exploring friction-based fixation methods led us to a solution in which the cables are looped inside the structure. As shown in Fig 2E, by looping a cable into a cross-shaped groove in the transversal plane of the segment and bending both its ends 90 degrees in the pulling direction, we obtain two independent actuation cables positioned at an angle of 90 degrees and connected in a sturdy cable fixation point (Fig 2D). Fixating four actuation cables per segment was realized by adding two cable fixation points on top of each other, rotated over 180 degrees. The total height of the resulting fixation module was 3 mm, leading to a total segment length of 12 mm. For our prototype, we decided to use an outer diameter of 8 mm.

## Proof of concept—HelicoFlex

**Fig 4:**
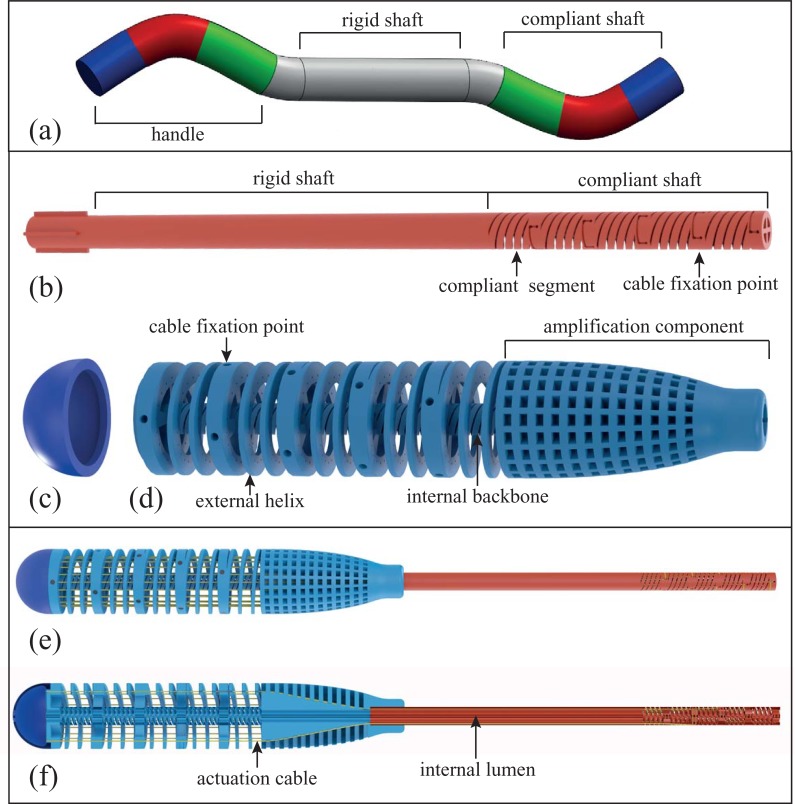
Phases in the development of the HelicoFlex. (a) chosen serial control method in which each colored segment of the compliant handle controls the corresponding mirrored segment of the compliant shaft; (b) compliant shaft connected to rigid shaft in which a compliant segment and a cable fixation point are highlighted; (c) end cap in which the loose ends of the actuation cables are stored; (d) compliant handle in which the external helix, the internal backbone, a cable fixation point at the handle side and the amplification component, to gently increase the distance between the cables from Ø 6.5 mm in the shaft to Ø 22 mm in the handle, are highlighted; (e) assembly; and (f) cross section, showing one of the internal lumens and two actuation cables.

After characterizing a single 3D printed segment of the compliant shaft, we designed the entire HelicoFlex (Fig 4). The instrument is composed of only three components: a compliant shaft and a compliant handle with a rigid shaft in between. We designed a handle with a compliant structure equivalent to the compliant shaft. The compliant shaft and the handle were connected using cables that actuate the device with a serial control method, described by Fan *et al*. [27], in which the compliant shaft mirrors the movement of the compliant handle (Fig 4A).

The *compliant shaft* of the device is composed out of compliant segments (Ø 8 mm, length 12 mm) stacked on top of each other to create a modular shaft in which the number of segments can be changed according to the number of DOF required. We decided for a shaft with five compliant segments, resulting in a total of 10 DOF at a length of 60 mm (Fig 4B). Each compliant segment has a backbone of Ø 1 mm and is actuated by four cables that are fixed at the segment location, as shown in Fig 2E, and run along the compliant shaft in a cable-ring configuration. Each segment is twisted around its heartline over 18 degrees as compared to the previous segment in order to avoid overlapping of the 20 parallel-running cables.

The *rigid shaft* (Ø 8 mm, 120 mm long) is printed in one part together with the compliant shaft and guides the cables from the compliant shaft to the handle through dedicated grooves running along its entire length (Fig 4B).

The *compliant handle* is connected to the rigid shaft by means of a press-fit mechanism, and its design is based on a large version of the compliant shaft (Fig 4D). The handle contains an inner backbone and an outer helical structure. The inner backbone has a structure similar to the compliant shaft, containing five segments with a diameter of 8 mm and a length of 18.5 mm. The outer helical structure of the handle has an outer diameter of 29 mm and contains holes through which the cables run. Running the cables through the handle at a larger diameter than in the compliant shaft creates not only additional space for precise fixation of the cables but also creates an amplification factor between the handle and the compliant shaft. Assuming that there is no friction, no play, no compression of the printed parts, and no stretching of the cables, the amplification factor can be calculated using the following equation:

$$\gamma =\beta (D_{\mathrm{handle}}/D_{\mathrm{shaft}}),$$ (1)

where *γ* is the desired bending angle of the tip, β is the corresponding bending angle of the handle, D_handle_ is the diameter of the cable ring in the compliant handle, and D_shaft_ is the diameter of the cable ring in the compliant shaft. In order to guide the cables smoothly from the shaft to the handle, an amplification component was designed at the distal side of the handle to gently amplify the cable distance from 6.5 mm to 22 mm. The amplification component guides the cables smoothly through partly covered S-shaped grooves while avoiding buckling. The amplification component is covered by a hive-inspired structure with holes. The hive structure facilitates the 3D printing process by enabling precise printing of cable grooves, avoiding clogging (Fig 4F).

In the handle, cables were fixated via dog point set screws to enable easy fine-tuning. The ends of all cables were collected and stored inside the end cap (Fig 4C), attached to the handle by a press-fit mechanism. Four lumens with a diameter of 1.75 mm run through the entire device to enable the insertion of thin, flexible instruments such as biopsy forceps, or thin, fiberoptic endoscopes to visualize the site of interest.

## Fabrication

Considering the small size of some of HelicoFlex’s features, we selected the AM technology taking into account the resolution achievable by the printer. A Perfactory® 4 Mini XL (EnvisionTec GmbH, Gladbeck, Germany), with a layer height in the vertical z-axis of 25 μm, was used to fabricate all three parts of the device. The used printer is based on vat photopolymerization technology and uses the so-called Digital Light Processing (DLP) in which the combined work of a light source and a projector hardens the liquid resin layer by layer [36]. We printed our prototype using the R5 epoxy photopolymer resin (EnvisionTec GmbH, Gladbeck, Germany).

**Fig 5:**
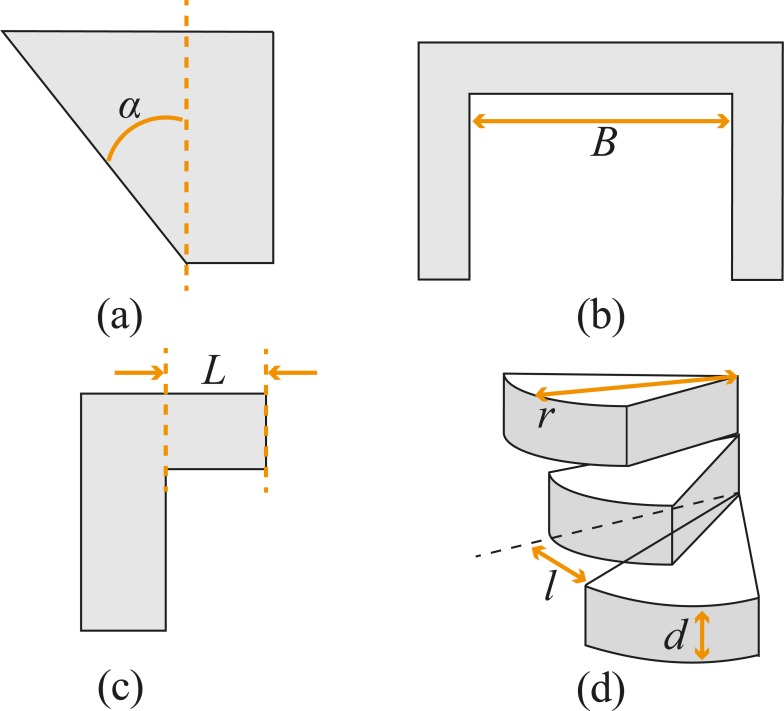
Rules to print without support. (a) reducing overhang angle *α*; (b) limiting the length of bridges *B*; (c) shortening overhanging structure *L*; (d) the rules applied in our helicoid: reducing the overhang angle by increasing the pitch or reducing radius *r* at a given layer thickness *d* will decrease the overhang *l* and improve the possibility of self-support.

Both the handle and the shaft were printed vertically with the long axis parallel to the vertical z-axis of the printer. As DLP printing technology requires overhanging structures to be printed with support material, this would require a rather elaborate post-processing step in the removal of support material within the detailed helical structure. A number of studies carried out in the field of additive manufacturing for support structures show, however, that support structures are not always necessary providing that the length of overhanging layers is limited [37][38][39][40][41]. Following these studies, we decided to print the segments of the compliant shaft without support, using three general rules (Fig 5): reducing the overhang angle (*α*), limiting the length of bridges (*B*), and shortening overhanging structures (*L*).

The *overhang angle* is the angle between printed layers (i.e., the critical angle). Increasing the layer thickness while keeping the number of revolutions of the helicoid as well as the length of the segment equal, increases the overhang angle between two layers and thus the need for support. Therefore, by keeping the layer thickness small (25 μm) and the pitch of the helix equal to its height, the overhang angle is kept narrow, and the helicoids can support themselves. *Bridges* define the distance between two unconnected points; limiting the length of the bridges avoids the use of support material. The role of the amplification component is to guide the cables through curved grooves. However, using solid material in such a long element would clog the grooves. Therefore, the hive structure was used to create short grooves, while at the same time avoiding long bridges that would have been created if rings had been used as cable guidance during the vertical 3D printing process. *Overhanging structures* are shapes that stick out horizontally parallel to the building platform. In our design, combining helicoids with a central backbone allows the compliant shaft to be printed without extra support due to the constant presence of support (the backbone) in the structure [42].

Applying these rules, we printed the entire compliant shaft without any support in a single printing run. Printing without support led to a strong reduction in post-processing time with an additional advantage that eliminating support material from the printing process resulted in smooth surfaces without debris that could cause malfunctioning of the mechanism, especially in elements with a small size.

Printing the handle and the shaft vertically allowed the grooves for the cables to remain open along the entire length of the shaft and the handle. The handle and the shaft were printed all together in 26 hours. After the printing, all the parts were placed in an ultrasonic cleaner for a few minutes.

**Fig 6:**
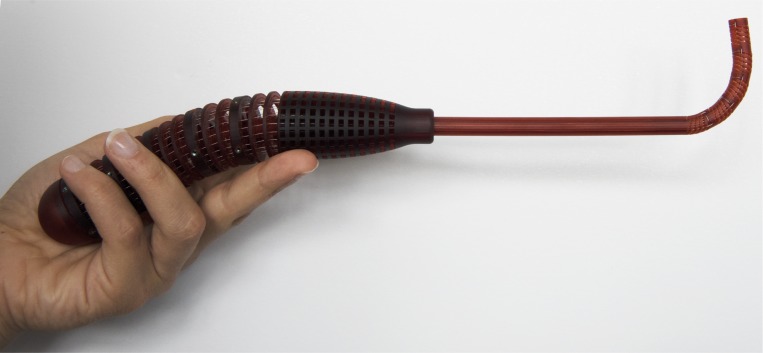
The assembled HelicoFlex prototype showing a single curved shape.

In order to control the compliant shaft, each segment must be coupled with the corresponding segment in the handle using the corresponding actuation cables. Running all 20 cables (stainless-steel Ø 0.2 mm) through the shaft and the handle took around 5 hours. In the handle, the cables were fixated by dog point set screws to allow fine-tuning in this prototype. Although it was possible to tap directly into the 3D printed material, this could have created points of brittleness in the handle. We, therefore, decided to place threaded inserts that allow the cable to be fine-tuned multiple times. During the assembly, the instrument was vertically placed and each cable was straightened by weights of 3 grams before fixation in the handle. The entire HelicoFlex prototype is shown in Fig 6.

## HelicoFlex performance

### Steering evaluation

**Fig 7:**
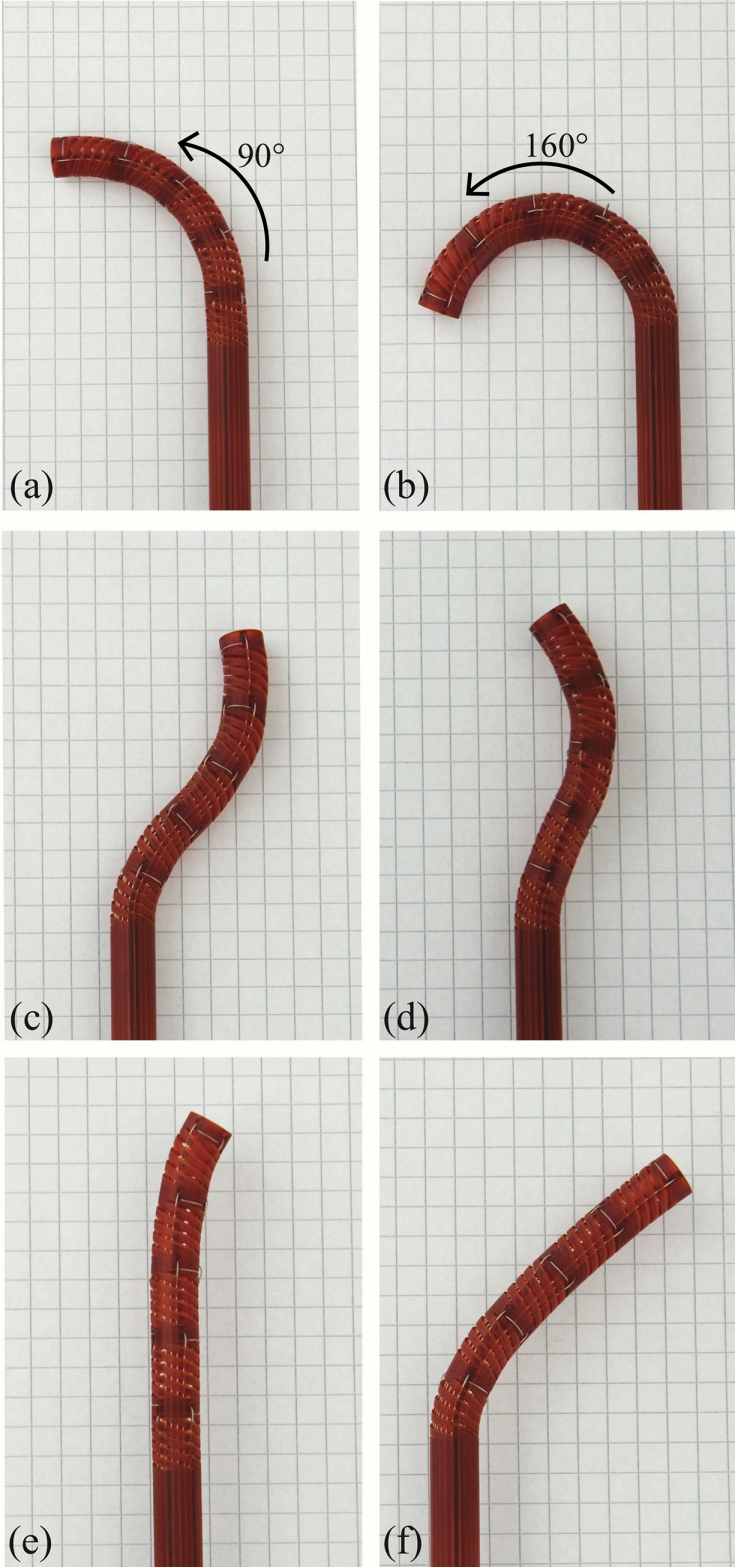
HelicoFlex compliant shaft bent in various shapes. (a) 90 degrees single curved shape; (b) 160 degrees single curved shape, reaching the maximum bending angle of the compliant shaft in performing a C-shape; (c) double curved with equal radii; (d) double curved with different radii; e) only the distal segment controlled; and (f) only the proximal segment controlled.

Simulating navigation through tortuous paths, the tip was moved along curves with different radii and shapes to evaluate the performance of the device, Fig 7. The prototype was able to perform single curved shapes with different angles (Fig 7A and 7B) and double curved shapes with different radii (Fig 7C and 7D). Moreover, the device allowed controlling each segment individually (Fig 7E and 7F). At an angle of 160 degrees, the maximum bending angle of the compliant shaft was reached because the outsides of the helicoids touched each other in the inner bend (see Fig 3).

**Fig 8:**
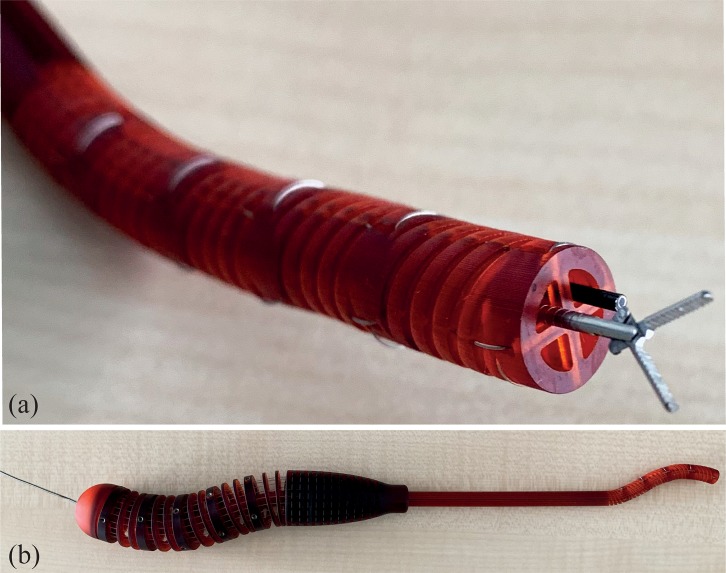
Pictures of the HelicoFlex prototype. (a) close-up of the compliant shaft of the HelicoFlex with a flexible biopsy forceps in one lumen and a flexible fiber optics endoscope in the second; (b) bendable metal rod inserted into one of the lumens to create a certain shape in the compliant shaft and keep it in position.

Besides moving the device over different angles and shapes, we also evaluated the possibility of using the internal lumen. The device allows the insertion of a flexible fiberoptic endoscope into one of its four lumens while leaving the other three lumens free for the insertion of multiple surgical instruments, such as a biopsy forceps, as shown in Fig 8A. Moreover, a bendable rod can be placed inside one of the lumens of the handle and can be shaped to hold the desired position (Fig 8B). The fluid motion, as well as the easy maneuverability of the HelicoFlex prototype, can be seen in the video attached to this paper (S1 Video).

### Payload test

For the instrument to be useful in a surgical setting, it must be able to withstand external loading. The bending stiffness of the HelicoFlex was therefore measured at the tip of the shaft. The shaft was tested in three different poses: straight, 90 degrees single curved, and in a double curved shape. To ensure that the poses were maintained during testing, 3D printed blocks were manufactured to properly constrain the handle in the required pose.

**Fig 9:**
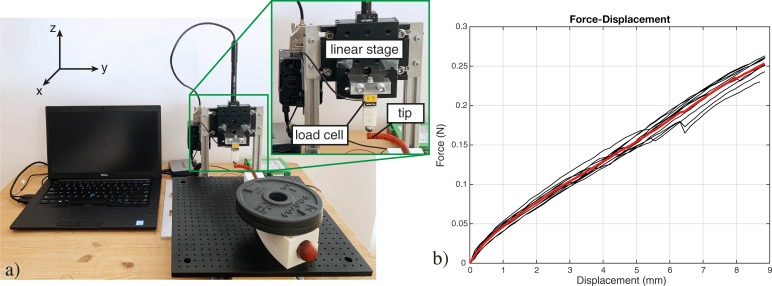
Setup of the payload test and an example of data acquired for the vertical direction. a) In the close-up different parts of the setup are highlighted. b) The experimental results for prototype 1 in single curved shape. The red line in the plot represents the averaged data.

The setup consisted of a load cell (S-Beam LSB 200 FUTEK Advanced Sensor Technology Inc., CA, USA, controlled by a custom-made LabView script) mounted on a linear stage (Thorlabs PT1/M-Z8, with additional KDC101 controllers, controlled by Thorlabs Kinesis software). The linear stage drove the vertical displacement of the load cell with a low, constant speed of 0.5 mm/s, as to induce a defined displacement of the prototype shaft. The load cell measured the generated tip force as a result of the induced displacement and the elasticity of the shaft. To ensure a precise and consistent point contact with the tip, a steel ball (Ø 10 mm) was screwed on the load cell. The prototype was placed perpendicularly to the linear stage movement (Fig 9A).

**Fig 10:**
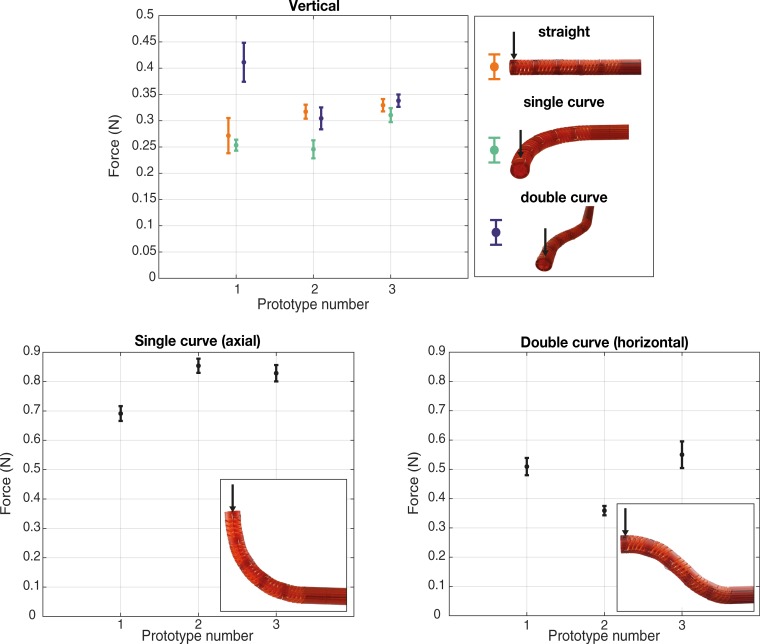
Plots of the average and the standard deviation of the peak-force for each different pose and prototype tested. The black arrows in the pictures indicate the direction of the displacement.

For the single and the double curved shapes, the force was measured in two different directions: vertical (perpendicular to the plane of the curved shape) and axial (along the prototype main axis) for the single curved shape, and vertical and horizontal (in the plane of the curved shape) for the double curved shape (Fig 10). For the straight shape, the force was measured in the vertical direction. Different directions were achieved by simply rotating the prototype. Due to possible variations given by the 3D printing process and the post assembly, three prototypes were tested in each pose, and each measurement was repeated ten times. Data were acquired from the moment the load cell touched the shaft tip up to a displacement of 9 mm and analyzed using a MatlabR2020a script (Fig 9B).

Fig 10 shows the average peak force in the tested configurations for three prototypes in each pose. The bending stiffness in the vertical direction for the straight shape was in the range of 0.030–0.035 N/mm. For the single curved shape, the bending stiffness in the vertical direction was in the range of 0.026–0.034 N/mm, while higher bending stiffness was measured in the axial direction (0.077–0.095 N/mm). For the double curved shape, the measured bending stiffness ranged between 0.033–0.045 N/mm and 0.039–0.061 N/mm vertical and horizontal direction, respectively.

## Actuation force test

HelicoFlex was designed as a manually powered device. To evaluate the actuation force required from the user to bend the steerable shaft with and without load, a setup similar to the payload test was used. We analyzed the required force to bend the proximal segment of the handle. Due to its high flexibility, the handle bends under its own weight if not supported. Therefore, except for the proximal segment that was left free, the handle was supported with a 3D printed block and the amplification component was constrained to stabilize the prototype. A load cell was mounted on a linear stage. The linear stage allowed the vertical displacement of the load cell with a low, constant speed of 0.5mm/s. The prototype was perpendicularly placed with respect to the linear stage, with the proximal segment of the handle underneath the load cell. To ensure consistent contact with the proximal segment of the handle, a steel ball (Ø 10 mm) was screwed on the load cell. Data were acquired for a vertical displacement of 3 mm of the proximal segment of the handle. Three prototypes were each tested with three different loads applied at the tip of the steerable shaft: 0 g, 6 g, and 18 g. These loads were manufactured from aluminum (6 g) and brass (18 g) as to have the same volume, shape, and center of mass, but different weights. The measurements (three prototypes for three conditions) were repeated ten times for each condition (0 g, 6 g, 18 g). Data were analyzed using a MatlabR2020a script.

**Fig 11:**
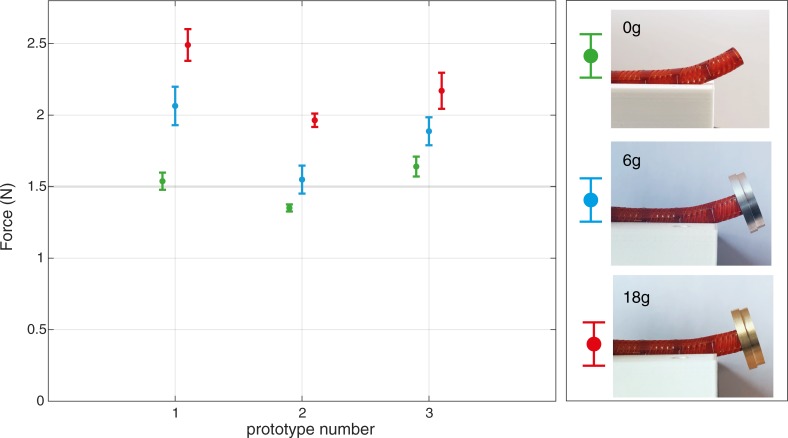
Peak force measured to bend the proximal segment of the handle with the tip loaded with 0 g, 6 g, and 18 g. The pictures on the right show the bending angle of the tip in the three conditions at the maximum proximal handle displacement (3mm).

Fig 11 shows the average peak force at 3 mm of displacement for the three conditions for each prototype. The peak force was in the range of 1.35–1.65 N, 1.55–2.00 N, and 1.95–2.50 N respectively for 0 g, 6 g, and 18 g applied. As expected, the force that the user must apply to the handle increases with the load carried by the tip. Moreover, the bending angle of the tip of the steerable shaft decreases as the load carried on the shaft increases: from the straight position, we measured 32 degrees for 0 g, 22 degrees for 6 g, and 18 degrees for 18 g.

## Discussion

In this work, we presented the world’s first fully 3D printed handheld multi-steerable instrument with five individually controlled compliant segments providing a total of 10 DOF. One of the requirements expressed in the “Design requirements” Section was the simplification of the assembly. In the conventional design of the EndoWrist, each part of the steerable shaft is designed for a single function as a result of which the device contains a significant number of parts that all have to be individually handled during the assembly phase, which requires a large amount of time and work. In order to greatly simplify the assembly, we integrated all the functionalities in the combined single shape of the helicoids, the backbone, and the cable fixation points, giving to the segment high axial and torsion stiffness and low bending stiffness with easy and reliable fixation of cables. In HelicoFlex, we integrated all these functionalities in a five-segmented compliant shaft, with a complex and unusual shape, yet 3D printed in only one printing job. HelicoFlex shows a fluid motion and easy maneuverability during the performance of multiple shapes allowing added multi-functionality due to the presence of the four lumens. The maximum bending angle that can be obtained is 160 degrees, which is much larger than the bending angle guaranteed by commercialized instruments such as the LaparoFlex (DEAM, Amsterdam, The Netherlands) or the Autonomy Laparo-Angle (Cambridge Endoscopic Devices, Framingham, MA, USA), which usually ranges over ± 60 degrees. The 8 mm diameter of the shaft was chosen equal to the diameter of most EndoWrist instruments and as a proof of concept in this paper. However, smaller diameters seem feasible without creating essential changes in the design.

The payload test showed that the bending stiffness of the steerable shaft is directly related to the direction of the external force. When the force was applied vertically, the bending stiffness ranged from 0.026 N/mm to 0.045 N/mm in the single curved and double curved shape, respectively. The bending stiffness increased when the force was applied on the plane of the curved shape, ranging between 0.039 and 0.061 N/mm (double curved shape). The maximum bending stiffness of almost 0.1 N/mm was measured when the external force was in the axial direction. To reach a bending stiffness suitable for surgical applications, it may be possible to increase the diameter of the internal backbone of the steerable shaft or the thickness of the helicoids. Also, it would be interesting to study how the material employed in the printing process affects the mechanical properties of the device. However, it is good to keep in mind that a balance has to be found between bending stiffness and more fatigue for the surgeon. The variance between the different prototypes is probably related to the 3D printing process and the minimal differences in manual straightening of the cables.

The general feeling while controlling the HelicoFlex is the easy maneuverability of the handle. Having a low bending stiffness reduces the forces required for actuation. We found that the actuation force increases linearly with the weight on the tip: increasing the load from 0 g to 6 g and from 0 g to 18 g required, respectively, 0.033–0.058 N and 0.033–0.047 N per added gram. Furthermore, the application of weight to the tip of the shaft influences the bending angle, which decreases with heavier loads. As currently designed, the prototype has a low bending stiffness not only in the steerable shaft but also in the handle, which bends under the weight of the shaft. Therefore, a higher bending stiffness would be desirable. Future research will focus on finding a good balance between the bending stiffness of the handle and the fatigue for the user.

The main goal of this work was to simplify the fabrication and assembly process of a multi-steerable device by using 3D printing technology to make it suited for disposable use. This led to a new type of continuous structure based on helicoids that could be printed without support. Moreover, we showed that it is possible to print an entire instrument out of one part, excluding the cables required for steering. In this prototype, we decided to use dog point set screws to fix the cables in the handle as this allows for easy fine-tuning of the prototype. Yet, we also experimented with cyanoacrylate glue that proved to be a fast and durable, much simpler alternative in the handle fixation (the cable fixation was tested tensioning the cable up to 1500 g for one hour with no sign of failure). The friction-based cable fixation in the compliant shaft required no other action than just looping the cables, which drastically decreased the assembly time. The entire device was printed in 26 hours, whereas threading and fixing the 20 cables took around 5 hours in this prototype. The handle and the shaft were printed with a layer thickness of 25 μm. Printing the same design with a layer thickness of 50 μm would drastically reduce the printing time to 13 hours, with only minor effects on the quality of the device. We found that the combination of helicoids with a continuum backbone limits the overhang angle between layers. In this way, we could avoid support material in the compliant shaft, which resulted in smoother surfaces without the presence of debris.

**Fig 12:**
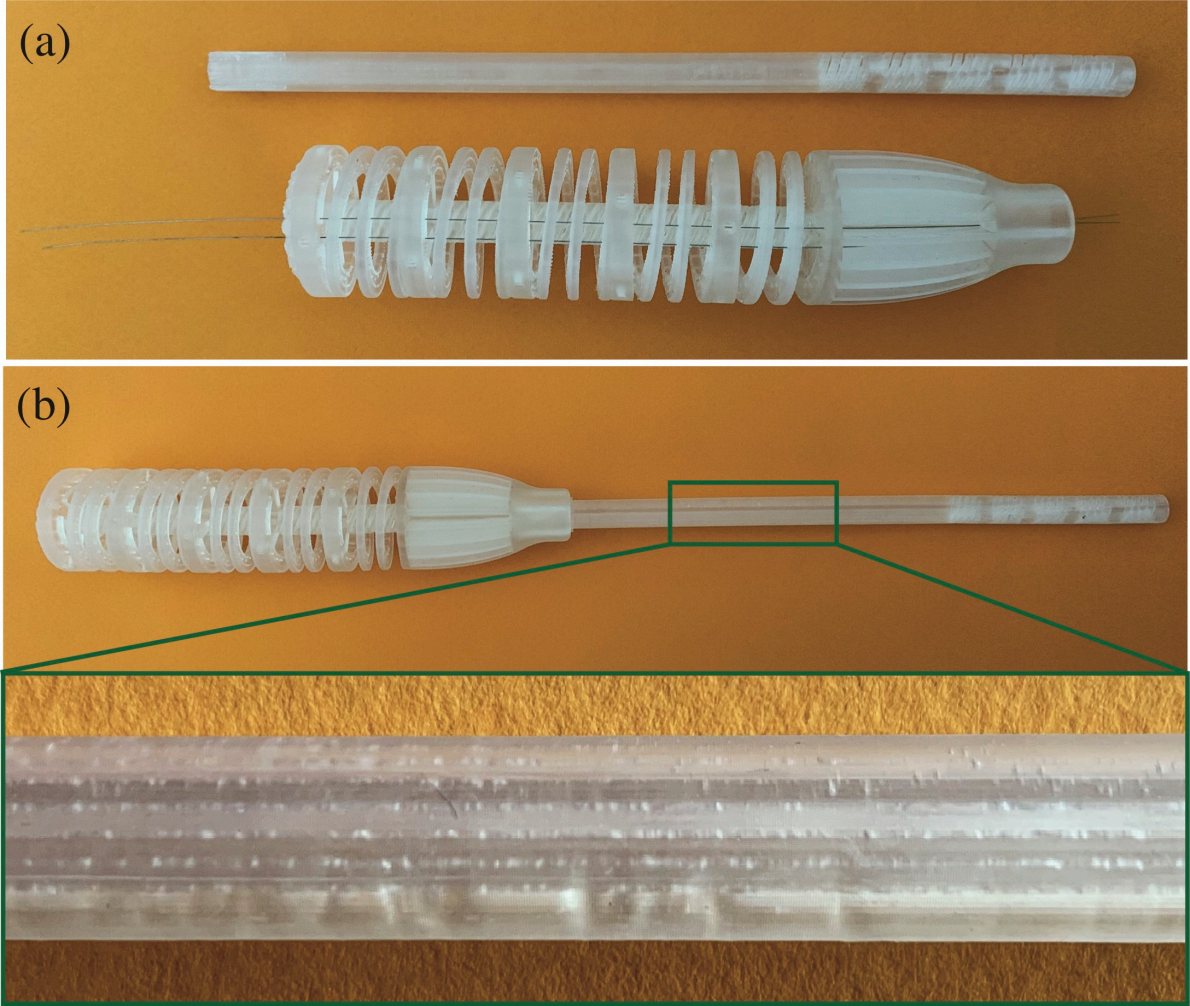
HelicoFlex printed with the E-Shell 600 biocompatible resin from (EnvisionTec GmbH, Gladbeck, Germany). (a) the shaft and the handle disassembled with two cables run through the handle; (b) the prototype assembled without cables with a close-up in which is visible how the grooves are clogged by liquid resin.

In our prototype, we used a non-biocompatible acrylic resin especially developed for prototyping. The use of this resin helped in the tuning phase to analyze and improve the design quality of the prototype. Biocompatible resins, such as E-Shell600, are provided by EnvisionTec as well. However, using this resin, results are decent but not yet sufficient due to the lower viscosity of this resin, which makes the cable grooves more difficult to be printed (Fig 12).

Using AM, the HelicoFlex can, in future surgical applications, be customized considering the surgery, the patients, and the surgeons’ needs, as well as made MRI-compatible by replacing steel cables by Dyneema. In the future, more tests to investigate the maneuverability of this new multi-steerable instrument in narrow environments will be performed as well as different materials and sizes, increasing our knowledge in this emerging new field of 3D printed medical devices.

**Fig 13:**
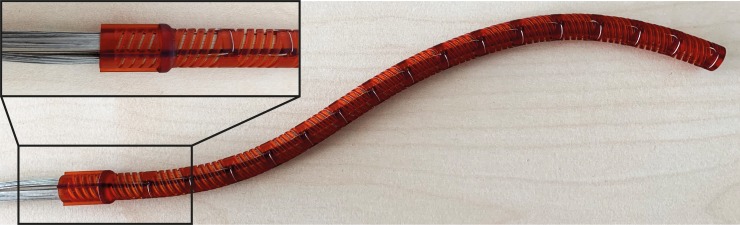
Example of 16 compliant segments combination with a close-up of the 64 actuation cables running through the Ø 8 mm shaft.

Using 3D printing is often considered a cheaper method of fabrication as compared to conventional manufacturing. However, it can be accounted as such only in specific cases (i.e., the production of complex devices that cannot easily be manufactured of molded conventionally) and if the necessary fine-tuning and testing time is taken into account (i.e., high initial costs for calibrating the settings of the printer, an expert who is able to tune and evaluate carefully how the design can be improved considering its use, long iteration phases to reach good results). Therefore, we believe that the real strength of AM is the capability of printing structures that are impossible to produce with conventional manufacturing and being able to integrate multiple functions into a single complex-shaped part. In a further elaboration, the number of segments can be increased, even more, reaching with the used cables and dimensions, a maximum length of 16 segments in a compliant shaft with 32 DOF (Fig 13). A multi-steerable structure this complex yet simple to assemble is a great step forward in the history of medical instrumentation.

## Conclusion

In this work, we have presented the first 3D printed multi-steerable device. We have shown that by adapting the design of a device to the fabrication capabilities of additive manufacturing, we have integrated multiple functionalities of different conventional elements into a single part, extensively decreasing the assembly time of a tendon-driven multi-steerable device for disposable medical use. The potential has been shown in a prototype: HelicoFlex. The handheld device, made out of three components, had five tendon-driven steerable segments for a total of 10 serially controlled DOF. HelicoFlex has shown a fluid motion and satisfactory results in performing different shapes. We have shown the high potential of additive manufacturing technology in building multi-steerable surgical instruments, limiting the number of components, and avoiding support material. HelicoFlex strives to contribute to the first generation of multi-steerable 3D printed instruments for MIS.

## Supporting information

S1 Video Video of the HelicoFlex while performing single- and double-curved paths in multiple directions. (MP4)
